# The Hidden Treasures of Preoperative Blood Assessment in Oral Cancer: A Potential Source of Biomarkers

**DOI:** 10.3390/cancers13174475

**Published:** 2021-09-05

**Authors:** Ana Caruntu, Liliana Moraru, Mihai Lupu, Lacramioara Taubner, Constantin Caruntu, Cristiana Tanase

**Affiliations:** 1 Department of Oral and Maxillofacial Surgery, Carol Davila Central Military Emergency Hospital, 010825 Bucharest, Romania; ana.caruntu@gmail.com (A.C.); liliana.moraru@yahoo.com (L.M.); 2 Department of Oral and Maxillofacial Surgery, Faculty of Dental Medicine, Titu Maiorescu University, 031593 Bucharest, Romania; 3 Dermatology Research Laboratory, Carol Davila University of Medicine and Pharmacy, 020021 Bucharest, Romania; lupu.g.mihai@gmail.com; 4 Clinical Laboratory, Carol Davila Central Military Emergency Hospital, 010825 Bucharest, Romania; corinataubner@yahoo.com; 5 Department of Physiology, Carol Davila University of Medicine and Pharmacy, 020021 Bucharest, Romania; 6 Department of Dermatology, Prof. N.C. Paulescu National Institute of Diabetes, Nutrition and Metabolic Diseases, 011233 Bucharest, Romania; 7 Biochemistry-Proteomics Department, Victor Babes National Institute of Pathology, 050096 Bucharest, Romania; cristianatp@yahoo.com; 8 Proteomics Department, Cajal Institute, Faculty of Medicine, Titu Maiorescu University, 031593 Bucharest, Romania

**Keywords:** oral squamous cell carcinoma, head and neck cancer, preoperative, fibrinogen, platelet-to-lymphocyte ratio, prognostic

## Abstract

**Simple Summary:**

In this study, we assess the prognostic potential of immune and inflammatory elements determined preoperatively in the peripheral blood of patients with oral squamous cell carcinoma (OSCC). Preoperative plasma fibrinogen (Fib) and platelet-to-lymphocyte ratio (PLR) show strong correlations with patients’ outcomes. Analyzed together, in a new parameter named Fibrinogen-PLR Algorithm (FiPLA), predictive power increases significantly. Clinicians can use this new, easy, cost-effective, and globally available tool for risk stratification of patients with OSCC, as early as from the moment of diagnosis.

**Abstract:**

(1) Background: Oral squamous cell carcinoma (OSCC) is a common malignancy, and the impact of immune and inflammatory mechanisms in its development and progression are of major interest. The aim of our study is to assess the prognostic potential of circulating immune and inflammatory elements determined preoperatively in patients with OSCC, as well as the development of a new compound parameter with predictive value. (2) Methods: We assessed preoperative fibrinogen (Fib) and the platelet-to-lymphocyte ratio (PLR) in 111 OSCC patients. Using a mathematic algorithm, we determined a composite parameter with cumulative information from Fib and PLR, named Fibrinogen-PLR Algorithm (FiPLA). Survival analysis, followed by bivariate and multivariate analyses, was subsequently conducted. (3) Results: Increased preoperative Fib and PLR levels were associated with poor outcome in OSCC (*p* = 0.0001 and *p* = 0.0015, respectively). Preoperative FiPLA values were also associated with poor patient survival (*p* < 0.0001). Multivariate analysis confirmed the independent prognostic role for FiPLA only (CI95% 1.232–67.770, *p* = 0.03), showing the superior predictive value of FiPLA compared to its individual components. (4) Conclusions: Preoperative assessments of circulating immune and inflammatory elements can provide high-quality prognostic information, and they represent valuable tools in clinical practice, facilitating the early risk stratification of patients with OSCC.

## 1. Introduction

Oral squamous cell carcinoma (OSCC) is a common malignancy worldwide, with a global incidence of 377.713 new cases in 2020 [1]. In 2018, Globocan reported that oral cancers were responsible for 2% of all malignancies and 1.9% of cancer-associated deaths [2]. Despite all forms of progress made in diagnosis and therapy for oral cancer, the age-adjusted mortality rates caused by this malignancy revealed an increasing trend during the last decade, with an average of 0.5% per year [3]. Currently, the only reliable prognostic element used worldwide in treatment-naïve patients is clinical TNM staging. Even though pathological TNM staging provides more accurate information, it is only available after therapy has been initiated and a patient has undergone surgical treatment [4]. Additional tumor characteristics that have confirmed a predictive value, such as the degree of differentiation, perineural invasion, lymph node micro-metastasis, or tumor immune infiltration, are available only after curative surgery has been performed and the entire specimen has undergone pathology assessment [5,6,7]. Thus, in the scientific world, there is a continuous quest for different elements with predictive potential that can increase the accuracy of disease characterization in terms of aggressiveness, response to therapy, and survival. Ideally, these biomarkers should be available as early as possible, even from the moment of diagnosis, and should be cost efficient for worldwide implementation, providing the advantage of optimal risk stratification and therapy selection before the initiation of any treatment. A wide variety of molecules that meet the criteria for biomarkers was investigated in scientific research conducted on OSCC; however, they were not introduced in standard clinical care due to the challenges raised by costs, the necessity of specific equipment, and specially trained personal [8,9,10,11,12].

Patients with OSCC undergo a general evaluation in their preparation for therapy, which usually includes blood tests. These peripheral blood tests allow for the assessment of the inflammatory and immune status of patients, two factors that are known to play major roles in most malignancies, including OSCC [13,14,15,16]. Cellular and molecular elements from the blood stream have revealed predictive attributes in patients with OSCC. Some studies have suggested the prognostic role of circulating cells assessed individually or in different ratios, such as the platelet-to-lymphocyte ratio (PLR) [17,18,19]. Acute phase inflammatory proteins have also been assessed for their predictive value in OSCC, with promising results [20,21]. However, in clinical practice it is challenging to interpret all this accessible but dispersed information. Thus, a unitary, easily applicable parameter available from the moment of diagnosis, which would incorporate different prognostic data within one numeric value and provide information regarding patient risk stratification, could be of great utility in clinical practice.

In our study, we aim to assess the predictive value of preoperative immune and inflammatory parameters in patients with OSCC with the development of a novel algorithm that can facilitate data interpretation for all clinicians in order to provide additional prognostic information to clinical TNM staging before the initiation of any curative therapy.

## 2. Materials and Methods

### 2.1. Patient Selection

We included patients with a confirmed diagnosis of OSCC who were subsequently treated in the department of Oral and Maxillofacial Surgery, “Carol Davila” Military University Emergency Hospital Bucharest. This study was carried out with the approval of the Local Ethics Committee from our hospital (No. 25/27 November 2017). All patients were newly diagnosed with OSCC and had not received any previous treatment. During the period of 2016–2019, in our department, we registered a total of 223 patients with OSCC who were considered for further eligibility in our study. Inclusion criteria were defined as follows: patients with operable disease, who received no previous treatment, with no history of other malignancies, and with complete follow-up data. Subjects with inoperable disease or other decompensated comorbidities, as well as patients who did not complete the treatment and patients with previous or concomitant neoplasms or incomplete medical records, were excluded from the study.

### 2.2. Preoperative Assessment

Within the preoperative work-up protocol, patients underwent clinical, imagistic, and general status assessments, including preoperative blood tests. Blood samples were collected at an interval of 1 to 5 days before surgery. Preoperative fibrinogen levels (Fib), as marker of systemic inflammatory status, and platelet-to-lymphocyte ratio (PLR), as a marker of systemic immune status, were selected for our study analysis. After preoperative workup, patients underwent radical surgical treatment with or without adjuvant chemo-/radiotherapy, in accordance with national oncological guidelines. All patients entered a follow-up program, with periodic visits and complete clinical and imagistic assessment.

### 2.3. Statistical Analysis

Statistical analysis was carried out with Prism 9 software (GraphPad) and SPSS version 23 (IBM). We used Shapiro–Wilk test to assess the normality of data distribution in our group. Data with parametric distribution were analyzed using Student’s *t*-test, while data with no Gaussian distribution were analyzed using Mann–Whitney test. Correlation analysis was carried out with chi-square or Fisher’s exact test. We selected two elements for further analysis: Fib and PLR, and we conducted a receiver operating characteristic (ROC) curve analysis. Cut-off values were determined using the Youden index method. Prognostic data, defined as disease specific survival (DSS), were calculated as the time interval from diagnosis to death caused by disease progression. Overall survival (OS) was defined as the time interval from diagnosis until the last follow-up, for all living patients, or until death by any other cause except the disease. Recurrence was defined as local, regional, or distant metastatic disease progression. Kaplan–Meier method was used for survival analysis, comparing results from log-rank test for each group. Univariate and multivariate analyses were subsequently performed. Statistical significance was considered to be *p* < 0.05.

## 3. Results

### 3.1. Patient Characteristics

From 223 subjects diagnosed with OSCC in our department between 2016 and 2019, 111 patients met the eligibility criteria and were included in this study. Primary tumors involved oral mucosa sites: tongue, floor of the mouth, gingiva, buccal, and palate mucosa. The mean age of the patients was 60.68 years old. Most of the patients were male (79%), with a male:female ratio of almost 4:1. Advanced disease at the time of diagnosis (stages TNM III and IVA) was found in 72% of patients. Exposure to classic risk factors: tobacco and alcohol, was confirmed in 72% and 57% of cases, respectively. Neck dissection, unilateral or bilateral, in tumors involving midline, was performed in 87 patients (78%), and lymph node metastasis was confirmed in 55 patients (63%). Perineural invasion was found in 22% of patients, while 9% had vascular invasion. Positive margins after tumor resection were present in 17 patients (15%). The mean follow-up time was 36 months (4 to 60 months). During the follow-up interval, disease recurrence was recorded in 40% of patients, and 34 patients (31%) died due to disease progression. Results are shown in Table 1.

### 3.2. Assessment of Preoperative Inflammatory and Immune Parameters in Peripheral Blood

Analysis of the preoperative peripheral blood values for fibrinogen (Fib), white blood cells count (WBC), lymphocyte count (Ly#), lymphocyte ratio (Ly%), platelet count (PLT), and platelet-to-lymphocyte ratio (PLR) revealed significant differences for several of these parameters in those patients who died due to disease progression compared to surviving patients. The results are presented in Table 2. Increased preoperative Fib levels were found in patients who died secondary to disease progression (*p* < 0.001), with a median (IQR) of 566.5 mg/dL in the deceased group compared to 480 mg/dL in the surviving group of patients. WBC, Ly#, Ly%, and PLT did not exhibit statistically significant differences between the two groups. However, the ratio between peripheral lymphocytes and platelets (PLR) was significantly lower in the surviving group of patients, with a median of 119.85 compared to the deceased group, which revealed a median of 150.73 for this parameter (*p* = 0.005).

### 3.3. Determination of Cut-Off Points for Fibrinogen (Fib) and Platelet/Lymphocyte Ratio (PLR)

**Figure 1:**
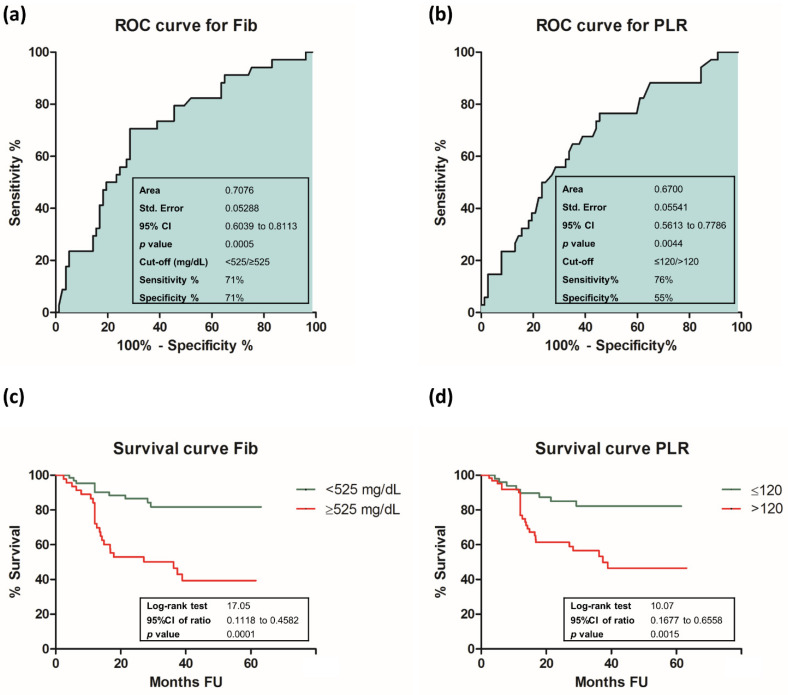
ROC and Survival curves for Fib and PLR: (**a**) ROC curve for Fib; (**b**) ROC curve for PLR; (**c**) survival curve for Fib; (**d**) survival curve for PLR. Statistical significance was considered at *p* < 0.05.

For further analysis, we selected Fib and PLR as preoperative markers of the systemic inflammatory and immune status in OSCC. ROC curve analysis revealed that the area under the curve (AUC) for preoperative Fib levels was 0.7076 (95% CI 0.6039 to 0.8113, *p* < 0.001), while for PLR, we calculated an AUC of 0.6700 (95% CI 0.5613 to 0.7786, *p* = 0.004). Using the Youden index method, we identified the optimal cut-off values for each parameter. The cut-off value for preoperative Fib was 525 mg/dL and it was 120 for PLR. Results are shown in Figure 1.

### 3.4. Correlation Analysis between Clinico-Pathological Features and Immuno-Inflammatory Peripheral Blood Parameters in OSCC

Based on the cut-off values, we divided our patients in two groups for each parameter and conducted a correlation analysis with clinico-pathological features in OSCC. Results are shown in Table 3. The two groups according to Fib were: Group 1, which included a total of 65 patients with Fib below cut-off and Group 2, which included 46 patients with Fib above cut-off. According to PLR cut-off value, there were the following: Group A, which included 49 patients with PLR ≤ 120 and Group B, which included 62 patients with PLR > 120. Our correlation analysis with clinico-pathological characteristics in OSCC revealed no significant correlations except for tumor recurrence, which was more frequently seen in patients with Fib and PLR levels above cut-off value (*p* < 0.001 and *p* = 0.002, respectively). In addition, smaller size tumors were associated with preoperative Fib levels below cut-off (*p* = 0.002).

### 3.5. Survival Analysis for Fibrinogen and Platelet/Lymphocyte Ratio in OSCC

Survival analysis showed that both Fib and PLR exhibited statistically significant correlations with patients’ outcomes. According to preoperative Fib levels, at the end of the follow-up interval, 85% of patients from Group 1 were alive compared to only 48% of patients from Group 2 (95% CI 0.1118 to 0.4582, *p* < 0.001). According to preoperative PLR values, survival analysis revealed that 84% of patients from Group A were alive compared to 58% of patients from Group B (95% CI 0.1677 to 0.6558, *p* = 0.002) Result are shown in Figure 1.

### 3.6. Assessment of Cumulative Prognostic Potential for Preoperative Fib and PLR

**Figure 2:**
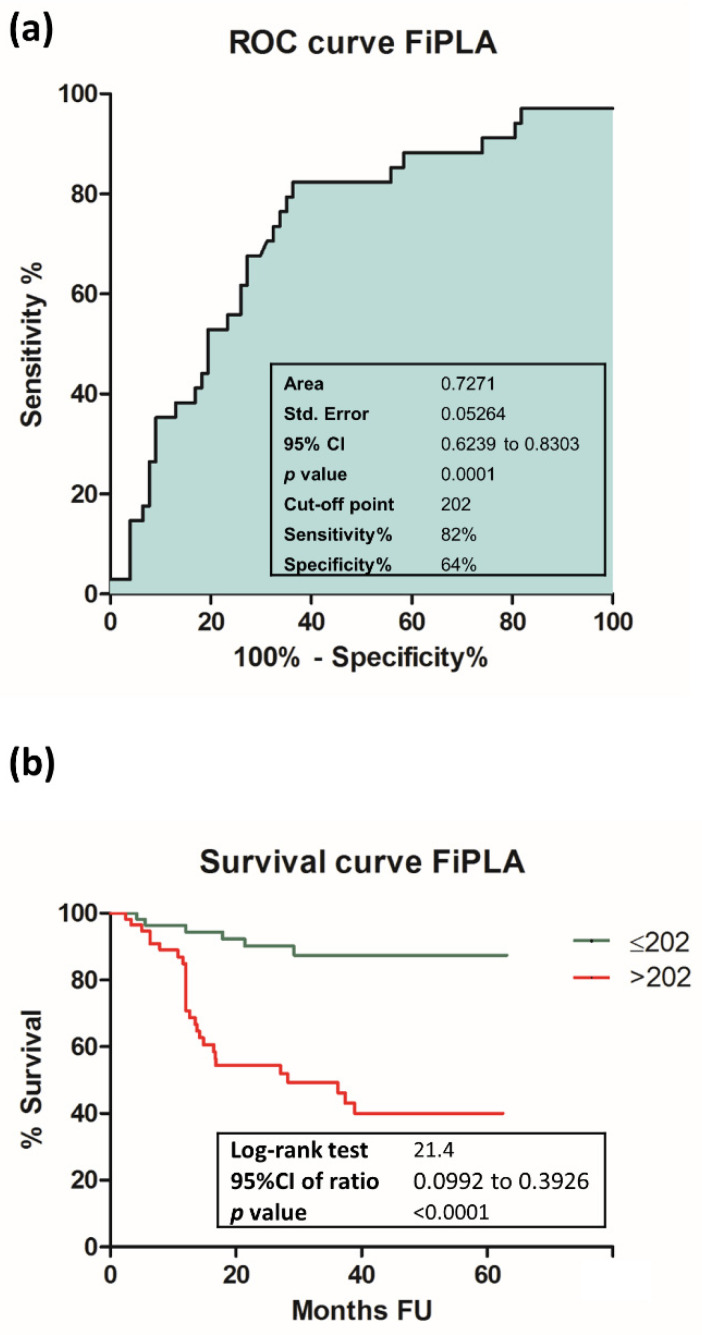
ROC and survival curves for FiPLA: (**a**) ROC curve for FiPLA; (**b**) survival curve for FiPLA. Statistical significance was considered at *p* < 0.05.

The cumulative power of Fib and PLR was calculated using, for each patient, percentage values from the previously determined cut-off levels for Fib and PLR, which were subsequently summed within a new parameter named Fibrinogen-PLR Algorithm (FiPLA). This calculation method allows for the mathematical addition of two otherwise distinct values—Fib, measured in mg/dL, and PLR, a cellular report—within a single numerical unit. The mean value of FiPLA was 222 (ranging between 123–523). ROC curve analysis for FiPLA resulted in an AUC of 0.7271 (95% CI 0.6239 to 0.8303, *p* < 0.001), with an increased value compared to its individual elements. The optimal cut-off point for FiPLA was 202 (sensitivity 82% and specificity 64%). Survival analysis based on preoperative FiPLA levels revealed that 89% of patients with FiPLA bellow cut-off and 51% of patients with FiPLA values above cut-off were alive at the end of the follow-up interval (95% CI 0.0992 to 0.3926, *p* < 0.001). Results are shown in Figure 2.

### 3.7. Multivariate Analysis for Fib, PLR and FiPLA in OSCC

A binomial logistic regression was performed to ascertain the effects of Fib, PLR, and FiPLA on the likelihood that subjects suffered death related to disease. The logistic regression model was statistically significant, χ^2^(3) = 26.004, *p* < 0.0005. The model explained 29.5% (Nagelkerke R^2^) of the variance in death related to disease and correctly classified 77.5% of cases (sensitivity 67.6%, specificity 81.8%, positive predictive value 62.16% and negative predictive value 85.13%). Of the three predictors, only two were statistically significant: Fib and FiPLA. Subjects with Fib above cut-off had 3.129-times higher odds of dying due to disease progression than those under cut-off (95% CI 1.149–8.520, *p* = 0.026). FiPLA revealed a superior predictive power, as subjects with FiPLA values over cut-off were 6.307-times more likely to die secondarily to disease than those under cut-off (95% CI 1.024–38.845, *p* = 0.047).

As certain clinico-pathological variables might affect the prediction pattern of these parameters, we ran a regression model controlling for age, TNM stage, smoking status, degree of differentiation, and surgical margin clearance. We excluded gender and recurrence from the analysis due to the high predominance of male sex and the complete separation of data for recurrence. The logistic regression model was statistically significant, χ2(11) = 39.021, *p* < 0.001. The model explained 41.8% (Nagelkerke R2) of the variance in death related to disease and correctly classified 77.5% of cases (sensitivity 55.9%, specificity 87%, positive predictive value 65.51% and negative predictive value 81.7%). Of the three predictors, only FiPLA maintained its statistical significance, independent of the controlling variables. Subjects with FiPLA over the cut-off value had 8.785-times higher odds of dying from disease progression (95% CI 1.133–68.115, *p* = 0.038).

## 4. Discussion

Controlling disease progression and improving patient survival have been the main goals in cancer research. These objectives led to an intense quest for biomarkers that have revolutionized cancer history in several types of malignancies [22,23]. However, despite all efforts to identify powerful biomarkers in OSCC, the survival of patients suffering from this disabling disease has not improved significantly in recent decades. Many simple and sophisticated, technologically challenging, and costly molecules have been investigated in OSCC, some of them displaying valid biomarker features [24,25]. In our study, we analyzed the predictive role of inflammatory and immune elements determined in peripheral blood in patients with operable OSCC. Our results revealed an enhanced systemic inflammatory status, expressed through elevated levels of fibrinogen found in most of the patients at the time of diagnosis. These values were significantly higher in patients with poor outcomes. Our results are in accordance with other studies that have confirmed the prognostic potential of fibrinogen in many types of malignancy [26,27,28]. Similar findings were reported in laryngeal, nasopharyngeal, oral, and oropharyngeal squamous cell carcinomas, where elevated preoperative levels of fibrinogen were associated with worse outcomes and resistance to therapy [29,30,31]. Increased fibrinogen values were correlated with an aggressive behavior in solid tumors, where deposits of fibrinogen organized in networks in the extracellular matrix facilitate the binding of growth factors, thus promoting tumor growth and invasion through angiogenesis and fibroblast proliferation [13]. Plasma fibrinogen, through an extravasation process, is the main source for these peritumor deposits. However, studies showed that some tumor cells have the ability to synthesize endogenous fibrinogen and release it in the peritumor environment [32]. This circulating glycoprotein provides protective assets for tumor cells released in the blood stream and facilitates their aggregation with platelets, preventing tumor cell clearance through immune effectors [33]. Analysis of the circulating blood cells conducted in our study group revealed no significant differences in relation to patient outcomes in OSCC. However, PLR was significantly higher in patients who died due to disease progression. The prognostic value of PLR is supported by other studies in different types of cancers, which have reported that increased PLR values act as a negative predictive factor in most malignancies [34]. Two meta-analyses conducted on OSCC and head and neck squamous cell carcinoma (HNSCC) patients reported similar results [18,35]. PLR is a composite parameter that encloses the information from two distinct cellular elements, platelets and lymphocytes, and the reported results suggest that the relationship between these two parameters have a major impact on patients’ survival. Platelets’ role in cancer progression is exerted through tumor cell–platelet aggregates that facilitate tumor cell survival and escape from immune clearance mechanisms [36]. Moreover, it has been shown that platelets support tumor metastasis and the systemic spread of cancers [37]. In the general population, elevated platelet counts, even within normal ranges, are associated with an increased incidence of cancers [38]. This suggests that platelets are involved not only in tumor progression, but also in the process of carcinogenesis. Lymphocytes—the second component of PLR—have been known for their major role in all phases of cancer development and progression. Lymphocytes, the main effectors of the antitumor immune response, exert their functions both at the tumor site and in the peripheral compartment. Extensive studies have been conducted during the last decades that have provided valuable information on the mechanisms that underlie and interfere with the systemic antitumor immune response, leading to one of the most important breakthroughs in cancer history—the discovery of immunotherapy [14,39,40]. Decreased lymphocyte densities in tumor tissue are associated with a poor outcome in OSCC [41,42]. However, for circulating lymphocytes, the results are not so consistent and divergent findings compared to our results have been reported [43]. It is interesting to observe that, even though the scientific literature lacks consistency in the reported results for peripheral blood platelet and lymphocyte counts regarding their prognostic potential in OSCC, when analyzed together as PLR, the situation changes completely, and most studies underscore their predictive role in OSCC [17,35]. The superior prognostic power of PLR compared to its individual components is probably due to the opposing roles of platelets and lymphocytes in cancer progression, and discrete changes in individual blood elements, which are not detectable in a routine assessment, become obvious when analyzed together in a unique parameter—PLR—that accumulates the information from each individual component. Based on this principle, we have further developed our analysis and evaluated the cumulative predictive potential of fibrinogen and PLR within the new parameter of FiPLA. To our knowledge, this is the first scientific report of an algorithm based on this principle of determination that is evaluated for its predictive power in cancer patients. Our analysis revealed that FiPLA proved its superior potential in detecting high-risk patients compared to its individual components. FiPLA can be considered a complex parameter that encompasses within one numeric value prognostic information provided by the inflammatory, immune, and coagulative systems in a cost-effective, easily applicable method that is available worldwide for the assessment of patients with OSCC. This new parameter can be used, in addition to clinical TNM staging, to stratify patients with OSCC based on prognostic risks, as early as from the moment of diagnosis. The limitations of our retrospective study are related to a single center location and a relatively reduced sample of patients; these are limitations that can be overcome by a large group in the future, as well as multicentric studies, both of which will evaluate the adequateness of cut-off values and validate these preliminary results.

## 5. Conclusions

Fibrinogen (Fib) and platelet-to-lymphocyte ratio (PLR) both act as prognostic factors for OSCC, and they demonstrate an increased predictive power when analyzed together within a composite parameter, the Fibrinogen-PLR Algorithm (FiPLA). In conclusion, we propose a new predictive tool that has several major advantages: a simple, cost-efficient, early available, reproducible method that is feasible for worldwide implementation and that can provide valuable prognostic information in patients with OSCC.

## Figures and Tables

**Table 1:** Descriptive statistics in OSCC.

Age, Years (Mean, SD)	60.68 (10.19)		
		No	%
Sex			
	Male	88	79%
	Female	23	21%
TNM stage *			
	I	6	5%
	II	25	23%
	III	22	20%
	IVA	58	52%
Smoking			
	Smokers	80	72%
	Nonsmokers	25	23%
	Missing	6	5%
Alcohol consumption			
	Drinkers	57	51%
	Nondrinkers	48	43%
	Missing	6	5%
T stage *			
	T1	13	12%
	T2	43	39%
	T3	31	28%
	T4	24	22%
Nodal status ^a^			
	pN0	32	37%
	pN+	55	63%
Histological differentiation			
	High	23	21%
	Intermediate	65	59%
	Low	23	21%
Perineural invasion			
	Confirmed	24	22%
	Not confirmed	87	78%
Vascular invasion			
	Confirmed	10	9%
	Not confirmed	101	91%
Resection margins			
	Positive	17	15%
	Negative	94	85%
Loco-regional recurrence			
	Present	44	40%
	Absent	67	60%
Follow-up			
	Alive	77	69%
	Dead due to disease	34	31%

^a^ neck dissection was performed in 87 patients; * AJCC, Eighth Edition.

**Table 2:** Preoperative inflammatory and immune parameters in peripheral blood.

Parameter	Survivors	Deceased	
	Mean/Median (SD/IQR)	Mean/Median (SD/IQR)	*p* Value
Fibrinogen, mg/dL	480 (145.5)	566.5 (143)	<0.001 ^a^
White blood cells, k/µL	8.42 (2.565)	8.285 (3.61)	0.919 ^a^
Lymphocytes, k/µL	2.043 (0.61)	1.83 (0.646)	0.164 ^b^
Ly, %	25.3 (9.5)	20.95 (12.325)	0.378 ^a^
Platelets, k/µL	255 (94)	278 (93.5)	0.108 ^a^
Platelet-to-lymphocyte ratio	119.85 (49.44)	150.73 (84.738)	0.005 ^a^

SD: standard deviation, IQR interquartile range, ^a^ Mann–Whitney test; statistical significance, ^b^ Student’s-*t* test, *p* < 0.05.

**Table 3:** Correlation analysis with clinico-pathological features in OSCC.

Variable		Fib		*p* Value	PLR		*p* Value
		<525	≥525		≤120	>120	
Sex				0.056			0.643
	Male	56	32		40	48	
	Female	9	14		9	14	
T stage				0.002			0.197
	T1	10	3		7	6	
	T2	30	13		21	22	
	T3	19	12		15	16	
	T4A	6	18		6	18	
Nodal status ^a^				0.824			0.999
	pN0	19	13		15	17	
	pN+	34	21		25	30	
TNM stage				0.635			0.677
	I	4	2		3	3	
	II	17	8		11	14	
	III	13	9		12	10	
	IVA	31	27		23	35	
Smoking				0.221			0.869
	Smokers	46	34		36	44	
	Nonsmokers	15	10		10	15	
	Missing	4	2		3	3	
Acohol consumption				0.863			0.693
	Drinkers	34	23		23	34	
	Nondrinkers	27	21		23	25	
	Missing	4	2		3	3	
Histological differentiation				0.253			0.358
	High	14	9		12	11	
	Intermediate	41	24		25	40	
	Low	10	13		12	11	
Perineural invasion				0.646			0.495
	Confirmed	13	11		9	15	
	Not confirmed	52	35		40	47	
Loco-regional recurrence				<0.001			0.002
	Present	15	29		11	33	
	Absent	50	17		38	29	

^a^ from a total of 87 neck dissections; statistical significance *p* < 0.05.

## Data Availability

The datasets used and/or analyzed during the present study are available from the corresponding author.
